# Metagenomic next-generation sequencing for identification of central nervous system pathogens in HIV-infected patients

**DOI:** 10.3389/fmicb.2022.1055996

**Published:** 2022-11-15

**Authors:** Yunqi Zhu, Wenxuan Zhao, Xihong Yang, Yuanyuan Zhang, Xiaoling Lin, Xing Weng, Yali Wang, Cong Cheng, Yun Chi, Hongxia Wei, Zhihang Peng, Zhiliang Hu

**Affiliations:** ^1^Department of Infectious Disease, The Second Hospital of Nanjing, Nanjing University of Chinese Medicine, Nanjing, China; ^2^Center for Global Health, School of Public Health, Nanjing Medical University, Nanjing, China; ^3^BGI Infection Pharmaceutical Technology, BGI-Shenzhen, Shenzhen, China; ^4^Clinical Laboratory of BGI Health, BGI-Shenzhen, Shenzhen, China; ^5^Clinical Infectious Disease Center of Nanjing, Nanjing, China; ^6^School of Public Health, Nanjing Medical University, Nanjing, China

**Keywords:** metagenomic next-generation sequencing, central nervous system, HIV, opportunistic infections, cerebrospinal fluid

## Abstract

Although considerable interest in metagenomic next-generation sequencing (mNGS) has been attracted in recent years, limited data are available regarding the performance of mNGS in HIV-associated central nervous system (CNS) infection. Here, we conducted a retrospectively analyzing of the cerebrospinal fluid (CSF) mNGS reports and other clinical data from 80 HIV-infected patients admitted to the Second Hospital of Nanjing, China from March, 2018 to March, 2022. In our study, CSF mNGS reported negative result, mono-infection, and mixed infection in 8.8, 36.2, and 55% of the patients, respectively. *Epstein–Barr virus* (EBV), positive in 52.5% of samples, was the most commonly reported pathogen, followed by *cytomegalovirus* (CMV), *John Cunningham virus* (JCV), *torque teno virus* (TTV), *cryptococcus neoformans* (CN), *toxoplasma Gondii* (TE), and *mycobacterium tuberculosis* (MTB). 76.2% of the EBV identification and 54.2% of the CMV identification were not considered clinically important, and relative less sequence reads were reported in the clinical unimportant identifications. The clinical importance of the presence of TTV in CSF was not clear. Detection of JCV, CN, or TE was 100% suggestive of specific CNS infection, however, 60% of the MTB reports were considered contamination. Moreover, of the 44 (55%) mixed infections reported by mNGS, only 4 (5%) were considered clinical important, and mNGS failed to identify one mixed infection. Additionally, except for MTB, CSF mNGS tended to have high sensitivity to identify the above-mentioned pathogens (almost with 100% sensitivity). Even all the diagnostic strategies were evaluated, the cause of neurological symptoms remained undetermined in 6 (7.5%) patients. Overall, our results suggest that mNGS is a very sensitive tool for detecting common opportunistic CNS pathogen in HIV-infected patients, although its performance in CNS tuberculosis is unsatisfactory. EBV and CMV are commonly detected by CSF mNGS, however, the threshold of a clinical important detection remains to be defined.

## Introduction

Central nervous system (CNS) opportunistic infection remains an important cause of morbidity and mortality in HIV-infected severely immunocompromised patients (Tan et al., 2012; Bowen et al., 2016; Thakur, 2020). Timely and accurately etiological diagnosis is crucial for improving the prognosis of CNS opportunistic infections. However, for many reasons, this goal is difficult to be reached. One of the diagnostic challenges is that more than 100 pathogens could cause CNS infections with the initial neurological manifestations being indistinguishable (Tunkel et al., 2008; Granerod et al., 2010; He et al., 2016). Due to sample volume restriction, it is unpractical to detect all the pathogens through traditional diagnostic methods, which are generally target-dependent and require a prior knowledge of the causative agents. Stepwise targeting the most possible causative pathogens may be an acceptable strategy. Nevertheless, the performance of this strategy strongly relies on the clinical experience of the treating physician. Even the most qualified physician may miss the diagnosis when the clinical features are atypical or when the patient is infected with a rare pathogen. Finally, shortage of commercial diagnostic kits for many opportunistic pathogens would further compromise the investigation of CNS infections in HIV-infected patients.

Fortunately, the limitations of traditional microbiological tests may be overcome by recently developed metagenomic next-generation sequencing (mNGS). This novel strategy identifies nucleic acids from clinical samples in a target-independent way, and thus could theoretically detect all the pathogens in a single run (Simner et al., 2018b; Gu et al., 2019). The clinical application of mNGS has attracted great research interest. Accumulating evidences have demonstrated that mNGS could assist with etiological diagnosis including but not limited to CNS infections, pulmonary infections, bloodstream infections, and bone infections(Wilson et al., 2019; Huang et al., 2020; Zhang et al., 2020; Chen et al., 2020b; Hogan et al., 2021; Jing et al., 2021; Zhu et al., 2022). Most of studies revealed that mNGS has sensitivity similar to or even better than specific PCR tests, however, the specificity varies depending on the samples used and the quality control for avoiding contamination (Miller and Chiu, 2021; Jin et al., 2022). Favorable specificity has been demonstrated in sterile samples, such as mNGS of the CSF, blood, and bone tissue samples (Jing et al., 2021; Miller and Chiu, 2021). However, the specificity for diagnosing pulmonary infections by mNGS of bronchoalveolar lavage fluid was poor (Wang et al., 2019; Chen Y. et al., 2021), probably due to the contamination of oral flora and the difficult in differentiation between colonization and invasive infection. The unbiased mNGS is especially useful for identification of rare pathogens, infections with atypical manifestations, and mixed infections (Hu et al., 2018; Wang et al., 2019; Fang et al., 2020; Chen et al., 2020a). Together, it is reasonable to hypothesize that mNGS would substantially improve the microbiological diagnosis of CNS infections in HIV-infected patients. Currently, the utility of mNGS in this field is still incompletely understood.

A recently study suggested that CSF mNGS may be a useful tool for the diagnosis of CNS infection among HIV-infected patients (Chen J. et al., 2021). The study focused on the impact of CSF mNGS results on the subsequent management decisions of the treating physicians (Chen J. et al., 2021). In our study on a population with more advanced immunodeficiency compared to the former study (Chen J. et al., 2021), we aimed to explore the diagnostic performance of mNGS for assisting etiological diagnosis of specific HIV-associated CNS infection. We would focus on the following questions: (1) which microorganisms were reported by the CSF mNGS in HIV-infected patients; (2) was the detection of the microorganisms a reflection of cross-contamination? (3) did those reported microorganisms lead to the neurological symptoms; and (4) did CSF mNGS miss clinical important etiological agents?

## Patients and method

### Patients

This study included 80 HIV-infected patients hospitalized at the department of infectious diseases in the second hospital of Nanjing, China from March 2018 to March 2022. The setting is a tertiary referral hospital and is a designated hospital that provides HIV care. The inclusion criteria for this study were hospitalized HIV patients: (1) with neurological symptoms; (2) CD4 count less than 200 cells/μl; and (3) having CSF mNGS reports. The patients were given conventional microbiological tests. In addition, CSF samples were sent for PMSeq-DNA, which is a commercial mNGS service targeting pathogens’ DNA sequences offered by BGI-Shenzhen (BGI, 2016; Hu et al., 2018; Zhu et al., 2022). Fresh CSF samples were transferred to the lab with dry ice. If immediate sample transportation was not possible, CSF samples were stored transiently at −80°C in a refrigerator until transportation. CSF samples were generally analyzed within 24 h of sample collection. For each CSF sample, BGI was blinded to the CSF laboratory tests, and gave a diagnostic mNGS report containing a list of pathogens detected as well as their corresponding sequence reads. Written informed consents, regarding the commercial mNGS service, were obtained from patients or their guardians. Retrospective analysis of those patients’ data was approved by the ethics committee of the second hospital of Nanjing (2020-LY-kt061).

### Metagenomic next-generation sequencing

Before nuclear acid extraction, CSF sample was mixed with enzyme and glass beads, and was vortexed vigorously at 2,800–3,200 rpm for 30 min. Then, total DNA was extracted from 300 μl CSF sample using TIANamp Micro DNA Kit (DP316, Tiangen Biotech). DNA libraries were constructed by DNA-fragmentation, end-repair, adapter-ligation, and PCR amplification. After quality control with Agilent 2100, libraries were sequenced by BGISEQ-50 platform. Low-quality sequence reads and sequence reads mapped to the human reference genome (hg19) were removed. The remaining data were classified by aligning to microbial genome databases covering bacteria, fungi, viruses, and parasites. The reference databases could be downloaded from NCBI^1^ containing 4,945 whole genome sequences of viral taxa, 6,350 bacterial genomes or scaffolds, 1,064 pathogenic fungi genomes, and sequences of 234 parasites associated with human diseases (Zhu et al., 2022).

### Data extraction and clinical interpretation

We collected the demographic, clinical, laboratory, and imaging data of the patients from an electronic health record system. The electronic report of CSF mNGS was provided by BGI-Shenzhen, and pathogen list and the sequence reads were extracted from each CSF mNGS report. The final diagnosis was made by the expert panel through evaluation of the all-available clinical data, including clinical features, conventional microbiological tests, mNGS result, and the response to treatment. The clinical interpretation of the mNGS report focused on: (1) did a positive detection necessitates antimicrobial treatment? and (2) did a negative report guaranteed the excluding of specific CNS infection?

### Statistical analysis

Categorical variables were described as frequencies and proportions. Continuous variables were expressed by medians with interquartile ranges (IQR). Comparisons among different groups were done using Mann–Whitney U test or Jonckheere-Terpstra test, when appropriate. 95% CIs for sensitivity, specificity, positive predictive value, and negative predictive value were calculated according to the Wilson intervals method described by Lawrence D. Brown in 2001 (Brown et al., 2001). Statistical analysis was done by using GraphPad Prism version 8.0 or R version 4.1.3.^2^ All reported *p* values were two-sided and the significance level was set at 0.05.

## Results

### Characteristics of the patients

The clinical, laboratory, and imaging characteristics of the patients were summarized in Table 1. The medium age of the 80 patients was 37 (IQR:31–49.0) yeas and the majority (90%) of the patients were male. The enrolled patients were severely immunocompromised with a median CD4 cell count of 29 (IQR:7–72) cells/μl and a medium HIV viral load of 94,500 (IQR:1,848–203,000) copies/ml. Thirty (37.7%) patients were on antiretroviral therapy, and viral suppression (<50 copies/ml) was achieved in nine of those patients. Three quarters of the patients had fever. 76 (95%) patients presented with at least one CNS symptom, whereas three patients underwent lumbar puncture for abnormalities on brain imaging screening, and one was examined for suspected brain metastases. The most common neurological symptom was headache, occurring in 41 (51.2%) patients, followed by confusion, decrease of muscle strength, and nausea. Most of the patients (62/80, 77.5%) had abnormal brain images.

**Table 1:** Clinical, laboratory and imaging characteristics of the patients.

**Variables**	**Characteristics**^**1**^
Age, years	37 (30.5–49.0)
Sex	
Male	72 (90.0)
Female	8 (10.0)
ART status	
Naive	50 (62.5)
ART ≤ 12 w	11 (13.8)
ART > 12 w	19 (23.8)
Fever	60 (75.0)
Headache	41 (51.2)
Confusion	24 (30.0)
Seizure	11 (13.7)
Dysuria	5 (6.2)
Nausea	22 (27.5)
Vomiting	20 (25.0)
Decrease of muscle strength	35 (43.7)
Neck stiffness	2 (2.5)
CD4 cell count, cells/μl	29 (7.0–72.0)
≤50	53 (66.3)
>50,and < 100	9 (11.3)
≥100	18 (22.5)
HIV viral load, copies/ml	94,500 (1848–203,000)
<50	10 (12.5)
Cerebrospinal fluid analysis	
White blood cell count, 10^6^/L	3 (1.0–15.0)
Proteins, mg/L	538 (405.0–900.0)
Glucose, mmol/L	2.5 (2.2–3.2)
Lactate dehydrogenase, IU/L	26.5 (17.0–42.3)
Adenosine deaminase, IU/L	3 (1.2–4.0)
Abnormal brain imaging	62 (77.5)

^1^Categorized variables were expressed as *n* (%) and continuous variables were described as medians (interquartile ranges).

### Pathogens detected by CSF mNGS

**Figure 1:**
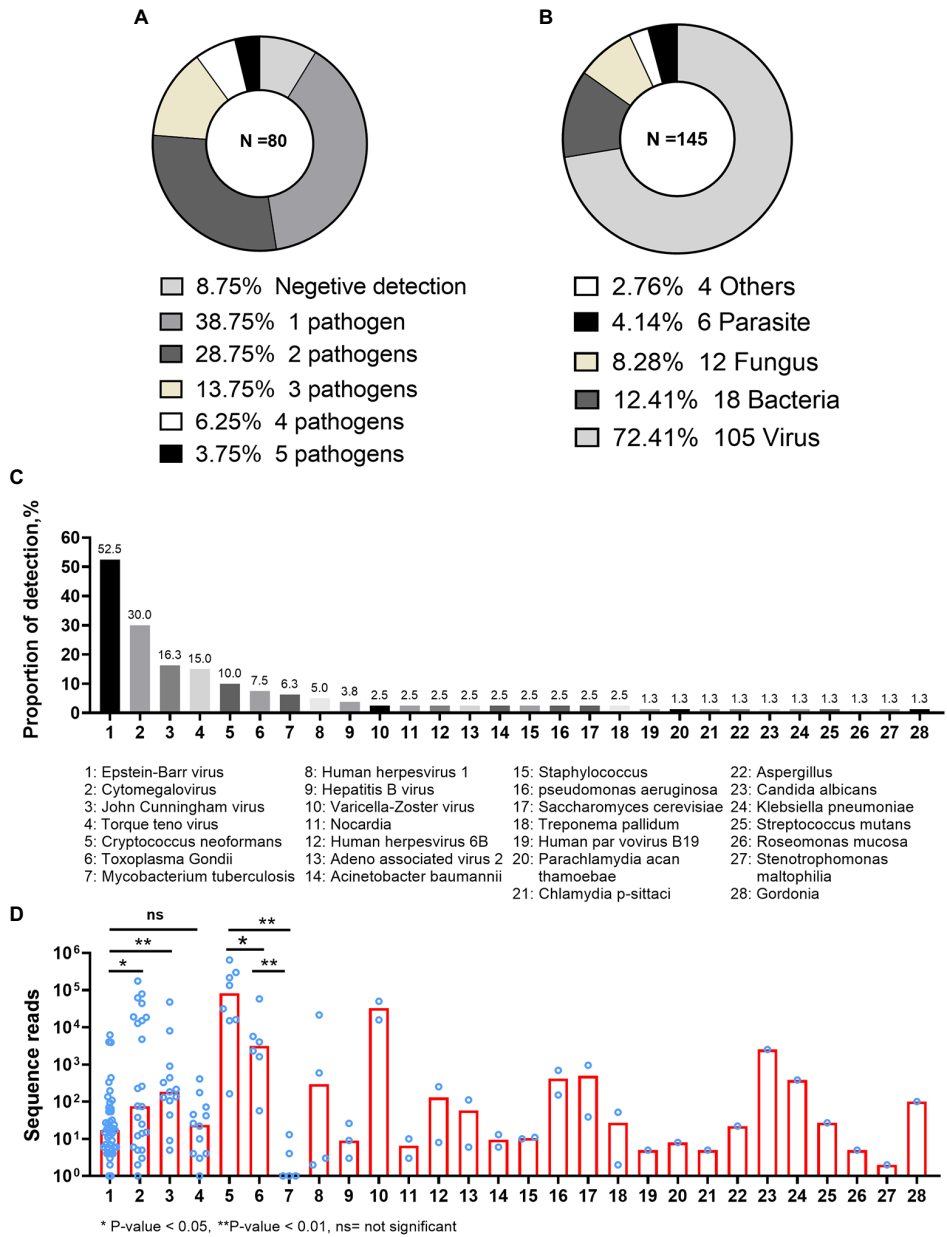
Pathogens detected by metagenomic next-generation sequencing. **(A)** The total number of pathogens presented per metagenomic next-generation sequencing (mNGS) report. **(B)** The proportions of every species reported by mNGS. The “Others” category included *Chlamydia psittaci*, *Parachlamydia acan thamoebae*, *and Treponema pallidum* (four positive identifications). **(C)** The proportion of each pathogen reported by mNGS. **(D)** The numbers of sequence reads reported by mNGS in different pathogens.

Of the 80 CSF mNGS tests, 7 (8.8%) gave negative report and 73 (91.2%) reported at least one pathogen. The presence of mixed pathogens was identified in 42(52.5%) patients (Figure 1A). Overall, 145 records of pathogen identification (from 28 pathogens) were reported by 80 mNGS reports. Of the 145 pathogen records, 108 (72.4%) belonged to the virus species (Figure 1B). *Epstein–Barr virus* (EBV) was the most common pathogen detected by CSF mNGS, being positive in 42 (52.5%) of the 80 CSF samples. The next six common pathogens were cytomegalovirus (CMV), *John Cunningham virus* (JCV), *Torque teno virus* (TTV), *Cryptococcus neoformans* (CN), *Toxoplasma Gondii* (TE), and *Mycobacterium tuberculosis* (MTB), which were detected in 24 (30.0%), 13 (16.3%), 12 (15.0%), 8 (10.0%), 6 (7.5%), and 5 (6.3%) of the 80 CSF samples, respectively (Figure 1C).

Variable numbers of sequence reads were reported by CSF mNGS, and seemed likely to be associated with the type of the pathogen (Figure 1D). Although EBV was the most common virus in the evaluated samples, it had much lower number of sequence reads as compared with CMV [median (IQR) of 18 (6–66) vs. 75 (8–17,756), *p* = 0.041] or JCV [median (IQR) of 18 (6–66) vs. 75 (8–17,756), *p* = 0.002]. The number of sequence reads was similar between EBV and TTV (*p* = 0.914; Figure 1D). As shown in Figure 1C, the fifth to seventh most common pathogens in the evaluated CSF samples were CN, TE, and MTB. Very low number of sequence reads of MTB [median (IQR) of 1 (1–9)] could be detected from CSF samples using mNGS. In contrast, much higher numbers of CN and TE sequence reads were detected from CSF samples, with median sequence numbers of 82,977 (IQR: 15,341–278,386) and 3,183 (IQR: 1,231–18,859; *p* = 0.002 and *p* = 0.004, respectively). Finally, although VZV was only detected in two patients, high numbers of VZV sequence reads were detected in both patients.

### Clinical evaluation of the detected pathogens in the mNGS reports

#### Detection of EBV and TTV

**Figure 2:**
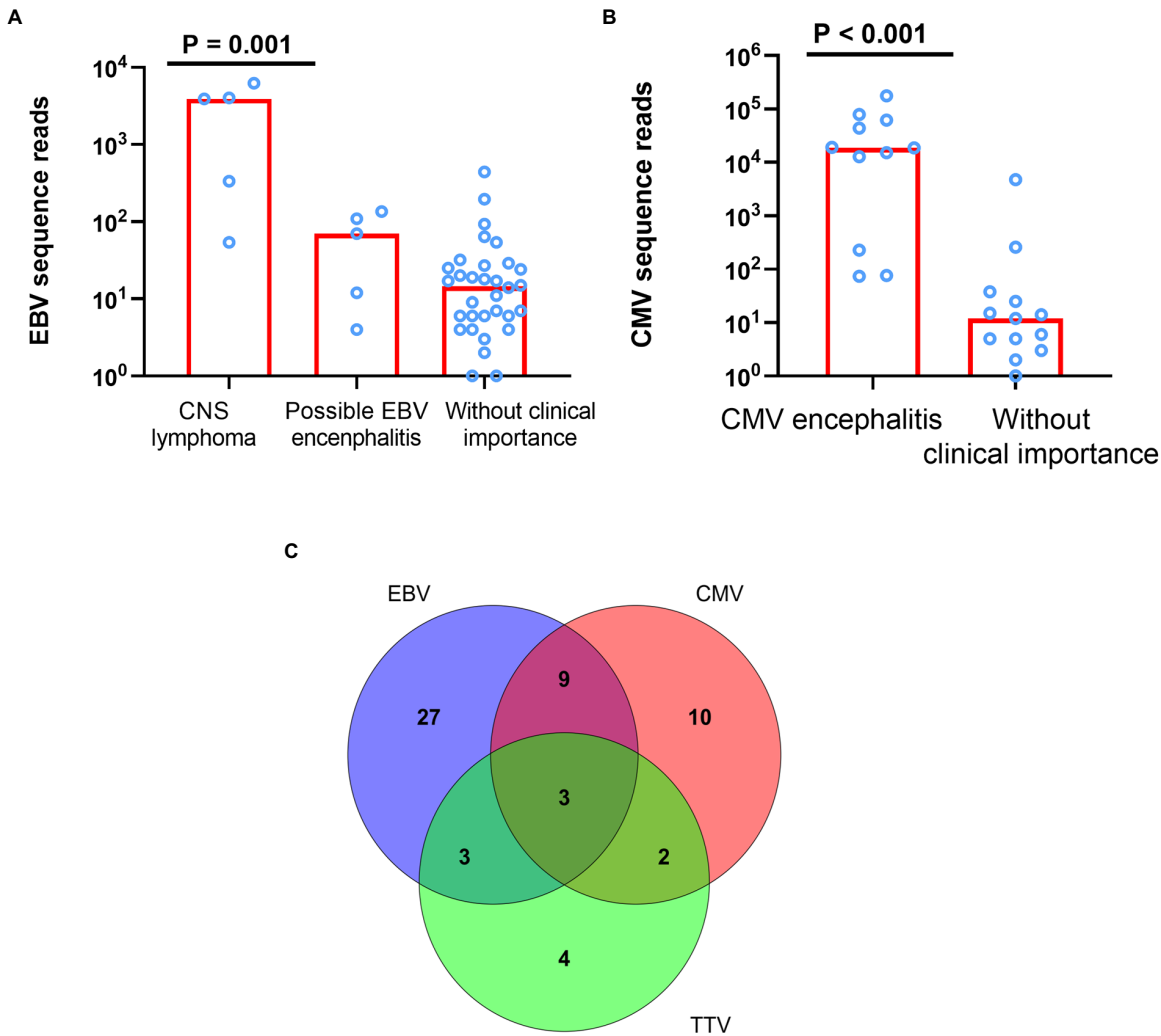
The difference of sequence reads between different clinical evaluation and mixed infection of EBV, CMV, and TTV. **(A)** The difference of EBV sequence reads among patients with CNS lymphoma, EBV encephalitis, and clinically unimportant EBV detections (*p* = 0.001). **(B)** The difference of CMV sequence reads among patients with CMV encephalitis and CMV detections without clinically unimportance (*p* < 0.001). **(C)** Mixed infection of EBV, CMV, and TTV. EBV denotes Epstein–Barr virus; CMV, cytomegalovirus; TTV, Torque teno virus.

Of the 42 patients tested positive for EBV DNA, 5 (11.9%) had CNS lymphoma (two histologically confirmed; three clinically suspected), 5 (11.9%) may possibly have EBV encephalitis (EBV was the only pathogen reported by mNGS), and 32 (76.2%) were not considered to have CNS diseases related to EBV infection. There was a trend of decreasing number of EBV sequence reads among patients with CNS lymphoma, EBV encephalitis, and clinically unimportant EBV detections (Jonckheere-Terpstra test, *p* = 0.001, Figure 2A). Of these 12 patients tested positive for TTV DNA, seven had co-existing CNS actively opportunistic infections (four CMV encephalitis, one PML, one cryptococcal meningitis, and one CNS tuberculosis) and one was considered as cryptococcal immune reconstitution syndrome. The clinical importance of the presence of TTV in CSF of HIV patients was not clear.

#### Detection of typical CNS opportunistic pathogens

Among the seven most common pathogens detected by CSF mNGS, CMV, JCV, CN, TE, and MTB were all typical CNS opportunistic pathogens (Bowen et al., 2016). Of the 24 patients tested positive for CMV DNA, 11 (45.8%) had CMV encephalitis (all had consistent clinical features; all CSF CMV PCR positive), and 13 (54.2%) were not consider to have CMV encephalitis. CMV sequence reads were much higher in patients with CMV encephalitis compared with those without CMV encephalitis [median (IQR) of 18,621(6,484–53,081) vs. 12 (5–25), *p* < 0.001, Figure 2B]. Of note, CSF mNGS falsely detected high number of CMV sequence reads (4,776) in a patient with transient headache. Since neurological symptom of this patient resolved without specific antiviral therapy, and CMV PCR tests of the plasma and CSF samples were both blow the limit of detection (<500 copies/ml), the detection of CMV sequences by mNGS was considered a false positive.

Unlike the detections of CMV and EBV, of which the clinical importance was associated with the number of sequences reads, all positive detections of JCV, CN or TE by CSF mNGS were clinically important. All 13 patients with JCV sequence reads and six patients with TE sequence reads have characteristic brain image abnormalities. Ten out of the 13 CSF samples with JCV sequence reads were sent for detection of JCV by PCR, of which 8 (80%) were detected positive. Serum anti-Toxoplasma IgG antibody was positive for the six patients with Toxoplasma Gondii sequence reads. All the eight patients with Cryptococcus neoformans sequence reads were confirmed to have cryptococcal meningitis using CSF cryptococcal antigen lateral flow assay.

Molecular detection of MTB in the CSF is an important strategy for diagnosing tuberculosis meningitis. The presence of MTB DNA in CSF samples is suggestive of CNS tuberculosis (Wilkinson et al., 2017). In the present study, five patients were tested positive for MTB by using mNGS with number of sequence reads ranged from 1 to 13. Only 2 (40%) cases were true CNS tuberculosis. The remaining three cases were not considered CNS tuberculosis because those patients did not have other evidences supporting MTB infection and the clinical condition resolved without anti-tuberculosis treatment. All those three patients had only trace MTB (one sequence read) reported by mNGS.

#### Detection of other pathogens

The remaining 21 pathogens account for 35 positive identifications in 22 patients (Figure 1C). Only five pathogens (HSV-1, VZV, *Treponema pallidum*, *Nocardia* spp., and *Chlamydophila psittaci*) had ever been considered the etiological agents of CNS infections with 11 positive identifications in total. Among these, only one of the two positive identifications of *Nocardia spp*. was considered contamination. The other 16 pathogens were either considered as non-CNS pathogens (such as HBV) or environmental pathogens.

#### Interpretation of mixed infections

**Figure 3:**
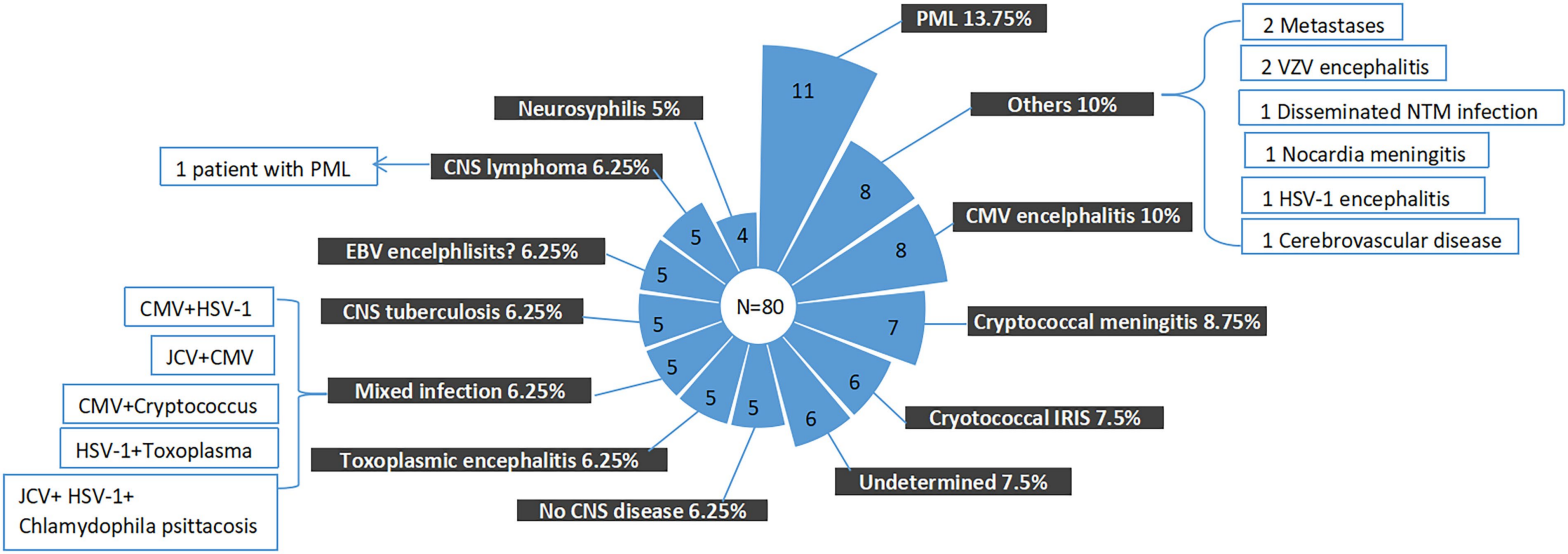
Final composite diagnoses of the study patients. EBV, Epstein–Barr virus; CMV, cytomegalovirus; PML, progressive multifocal leukoencephalopathy; CNS, central nervous system; JCV, John Cunningham virus; and HSV-1, herpes simplex virus 1.

As above-mentioned, CSF mNGS identified at least two pathogens in 44 (55%) samples. Of the seven most common pathogens detected by CSF mNGS, the presence of 4 (JCV, *Cryptococcus neoformans*, Toxoplasma *Gondii*, and MTB) in the CSF, regardless of the number of the sequence reads, were generally considered as clinically important, if not contaminated by other samples. Interpretation of the identification of EBV, CMV, and TTV was more complicated, since those viruses could persist in healthy individuals (Strassl et al., 2018; Wen et al., 2018).In our study, 58 (72.5%) CSF samples were positive for at least one of these three viruses and mixed infection of these viruses were detected in 17 (21.3%) CSF samples (Figure 2C). However, as described before, detection of those viruses in the CSF did not prove that they were the causes of neurological diseases. Mixed infections were only considered in 5 samples through final clinical diagnosis by experts panel, among which 1 was CMV and HSV-1 mixed infection, 1 was JCV, Chlamydophila *psittaci* and HSV-1 mixed infection, 1 was *Cryptococcus neoformans and* CMV mixed infection, 1 was *Toxoplasma Gondii* and HSV-1 mixed infection, and 1 was JCV and CMV mixed infection (Figure 3). mNGS correctly identified four of those five mixed infections, however, mNGS failed to detection JCV sequences in one patient deemed to have JCV and CMV mixed infection.

### Clinically important pathogens not in the mNGS report

**Figure 4:**
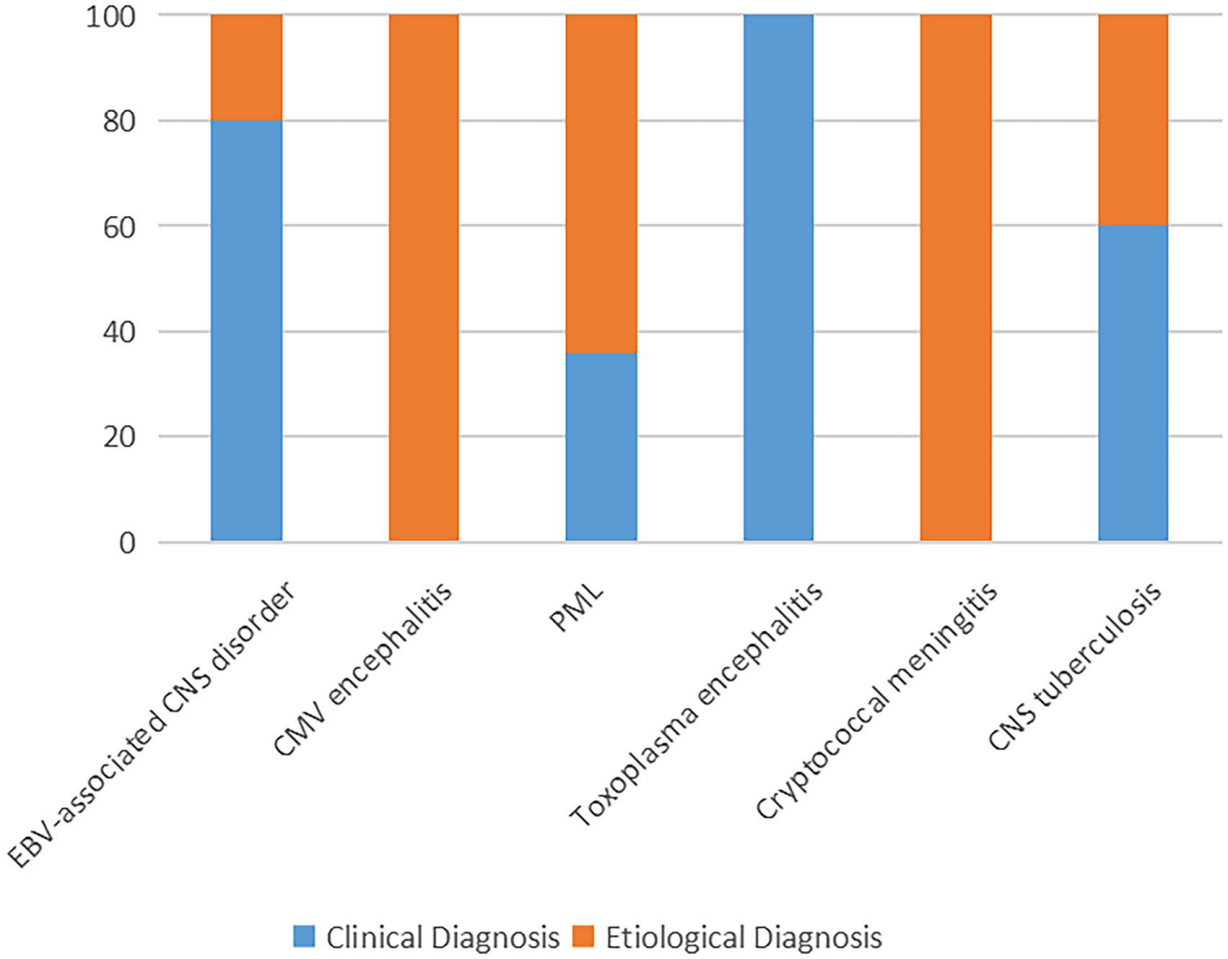
The proportion of final composite diagnoses. PML, progressive multifocal leukoencephalopathy; EBV, Epstein–Barr virus; CMV, cytomegalovirus; and CNS, central nervous system.

In clinical practice, the question that, to what extent, a negative report could exclude a specific infection is also important. In our study, even though mNGS was introduced as complement of the traditional microbiological methods, the causes of neurological symptoms remained undetermined in 6 (7.5%) patients. The finial composite diagnoses for the patients were shown in Figures 3, 4, and the corresponding biological parameters were listed in Table 2. Overall, CSF mNGS failed to identify the etiological agent in six patients, in which two were neurosyphilis, one was JCV infection, and three were CNS tuberculosis. Consequently, the calculated sensitivities of CSF mNGS for diagnosing of neurosyphilis, PML, and CNS tuberculosis were 50% (2/4), 92.9% (13/14), and 40% (2/5), respectively (Table 3; Supplemental Table 1). CSF mNGS was able to identify all the cases of clinical important EBV and CMV infection, toxoplasma encephalitis, and cryptococcal meningitis, which equal to a sensitivity of 100%.

**Table 2:** Microbiological parameters of final composite diagnosis.

**1. EBV-associated disorder (10 patients)**
Etiological diagnosis: two histologically confirmed CNS lymphoma
Clinical diagnosis: three clinical suspected CNS lymphoma and five EBV encephalitis.
Five CSF EBV PCR positive^1^
**2. CMV encephalitis (11 patients)**
Etiological diagnosis: all 11 patients CSF CMV PCR positive.
**3. Progressive multifocal leukoencephalopathy (14 patients)**
Etiological diagnosis: nine patients CSF JCV PCR positive^2^
Clinical diagnosis: five patients had typical clinical manifestation and consistent brain MRI features
**4. Toxoplasma encephalitis (six patients)**
Clinical diagnosis: all six patients had positive serum anti-Toxoplasma IgG and consistent brain MRI features.
**5. Cryptococcal meningitis (eight patients)**
Etiological diagnosis: all CSF cryptococcal antigen positive.
**6. CNS tuberculous (five patients)**
Etiological diagnosis: two CSF GeneXpert MTB/RIF positive^3^
Clinical diagnosis: three had confirmed tuberculosis in other organs and consistent brain MRI features that responded to antituberculosis treatment.

EBV, Epstein–Barr virus; CMV, cytomegalovirus; CNS, central nervous system; PCR，polymerase chain reaction. ^1^CSF EBV PCR was performed in six patients. ^2^CSF JCV PCR was performed in 11 patients. ^3^CSF GeneXpert MTB/RIF was performed in five patients.

**Table 3:** Diagnostic performance of CSF mNGS for common opportunistic CNS diseases^1^.

	**Sensitivity**	**Specificity**	**PPV**	**NPV**
	**(95%CI)**	**(95%CI)**	**(95%CI)**	**(95%CI)**
**EBV-associated CNS disorder**^**2**^	100	NA^3^	NA	100
	(72.25-100)			(90.11–100)
**CMV encephalitis**	100	NA	NA	100
	(74.12–100)			(92.87–100)
**PML**	92.86	100	100	98.36
	(68.53–98.73)	(93.98–100)	(77.19–100)	(91.28–99.71)
**CNS tuberculosis**	40	97.1	50	95.71
	(11.76–76.93)	(90.03–99.20)	(15.00–85.00)	(88.14–98.53)
**Toxoplasma encephalitis**	100	100	100	100
	(60.97–100)	(94.65–100)	(60.97–100)	(94.65–100)
**Cryptococcal meningitis**	100	100	100	100
	(67.56–100)	(94.50–100)	(67.56–100)	(94.50–100)

CSF, cerebrospinal fluid; mNGS, metagenomic next-generation sequencing; PPV, positive predictive value; NPV, negative predictive value; EBV, Epstein–Barr virus; CMV, cytomegalovirus; PML, progressive multifocal leukoencephalopathy; and CNS, central nervous system. ^1^For the purpose of illustration, all six patients with undetermined causes of neurological symptoms had been excluded from analysis. Finally, diagnostic performance of CSF mNGS was calculated from 74 samples. ^2^CNS lymphoma or EBV encephalitis. ^3^EBV and CMV were commonly detected by CSF mNGS. Some of the positive detections were not associated with CNS disorder, and also could not be considered as false positive either. Therefore, the specificity and PPV were not calculated.

## Discussion

HIV-infected patients are vulnerable to CNS opportunistic infections, the most common disease among which are cerebral toxoplasmosis, progressive multifocal leukoencephalopathy, tuberculous meningitis, cryptococcal meningitis, and cytomegalovirus encephalitis (Tan et al., 2012; Bowen et al., 2016; Thakur, 2020). CNS lymphoma, although not an infectious disease, has been associated with CNS EBV infection (Bossolasco et al., 2002; Wang et al., 2022). In the present study, those pathogens were also the major contributors to CNS disorders, suggesting that priority should be given to those pathogens when making a differential diagnosis. Importantly, the sensitivity of mNGS for identifying CNS virus infections (such as EBV, CMV, and JCV) was very high in our study. It is possible that, with continuing improvement of the methodology of mNGS, traditional viral detection tests may not be needed for a diagnostic purpose when mNGS has already been used for CSF pathogen detection. However, detection of viral sequences from CSF sample does not always confirm that the virus leads to CNS disorder. In our study, the clinical important detection of CMV and EBV was associated with the number of sequences reads reported by CSF mNGS.

Asymptomatic carry of TTV is very common in healthy individuals. Human pathogenicity of TTV has not been fully established (Takahashi et al., 1998; Hino and Miyata, 2007; Vasilyev et al., 2009; Focosi et al., 2020). The titer of TTV DNA load is inversely correlated with CD4 cell count in HIV-infected patients, and therefore could be a potential marker reflecting the degree of immune impairment (Shibayama et al., 2001; Schmidt et al., 2021). This virus has been identified in CSF samples from a proportion of patients with variable neurological complications, including HIV-infected patient with other coexisting CNS opportunistic infections (Pou et al., 2018; Simner et al., 2018a; Eibach et al., 2019; Neri et al., 2020; Perlejewski et al., 2020). In our study, seven out of the 12 patients test positive for TTV DNA had co-existing CNS opportunistic infections. It is difficult to prove whether TTV directly contribute to the neurological disorder or is just a mark of immune disturbance.

For a clinical perspective, detection of MTB, CN, and TE sequences in the CSF samples is generally regarded as etiological confirmation. In the present study, the sensitivity and specificity of CSF mNGS for diagnosing CNS CN and TE diseases both reach 100%. However, diagnostic performance of CSF mNGS for CNS TB was poor. The false positive detection of MTB sequences in CSF samples implies that mNGS could be also subjected to cross-contamination, a drawback that also has been encountered by traditional molecular diagnostic tests. In HIV-seronegative patients, the sensitivity of CSF mNGS for diagnosing tuberculosis meningitis ranges from 58.8 to 84.44% (Wang et al., 1993; Yan et al., 2020; Lin et al., 2021). Since MTB infection in HIV-infected patients generally leads to more disseminated diseases (Lee et al., 2000), the low sensitivity of mNGS for diagnosing HIV-associated CNS TB in our study is a little counter-intuitive. Future study is necessary to address whether this phenomenon is a reflection of less severe meningeal involvement in HIV-associated CNS tuberculosis. As a molecular diagnostic method, the sensitivity of mNGS for pathogen identification is expected to be closely related to the burden of that pathogen in clinical samples. Although CSF mNGS detected *Treponema pallidum* in two patients with neurological syphilis, the sensitivity for diagnosing neurological syphilis was not satisfactory. Taken together, although mNGS may be able to detect all kinds of pathogens from clinical samples, it has variable sensitivity for pathogen identification depending on the categories of the causative agents and the severity of the diseases.

Several reasons could explain why the detection of JCV, VN, and TE from CSF samples was much reliable for diagnosing the related CNS diseases compared with that of the CMV and EBV. It is well known that CMV and EBV could establish life-long persistence and replicate at low subclinical levels in reservoirs (Faulkner et al., 2000; Young et al., 2007; Goodrum, 2016). They may even be detected in peripheral blood mononuclear cells of the healthy blood donors (Larsson et al., 1998; Kanakry et al., 2016). The related clinical disorders tend to occur in patients with higher blood viral loads (Spector et al., 1998; Kanakry et al., 2016). In HIV-infected patients, detection of EBV DNA in CSF sample doses not prove EBV associated neurological diseases, although high CSF EBV DNA level is associated with CNS lymphoma (Wang et al., 2022). The low number of sequences CMV or EBV reads reported by CSF mNGS, in our study, may be a reflection of CMV or EBV persistence other than active diseases. Moreover, since both of the CMV and EBV are common human pathogens, the risk of cross contamination should not be underestimated. on the contrary, none of the JCV, VN, and TE have been thought to persist in healthy individuals, and the detection of pathogen DNA from sterile material generally proves clinical diseases (Luft and Chua, 2000; Lewinsohn et al., 2017; Donnelly et al., 2020). In addition, active infection with JCV, VN, or TE is relatively uncommon disease in China. The mNGS platform handle only limited samples in one run, and therefore the risk of cross contamination by JCV, VN, or TE is low.

Unbiased mNGS enables the detection of unexpected pathogens; however, it also detects environmental microbes contaminating the clinical samples. In our study, 28 pathogens were reported from 80 CSF samples. Most of the pathogens were not considered clinically important, and detection of those pathogens would complicate the clinical interpretation of the mNGS reports. In our study, if all the detected pathogens were considered clinically important, about half of our patients could be interpretated as CNS mixed infections. However, after discussion by the expert panel through which clinically unimportant detection and microbial contamination were evaluated, CNS disorders were considered to be related to mixed infection only in 5 (6.25%) patients. At present, there is no consensus standard for clinical interpretation of mNGS results. This process is not always objective and would inevitably introduce bias. For example, a recent study unexpectedly identified *Aspergillus* spp. from CSF samples in two HIV-infected patients (Chen J. et al., 2021). Although the two patients also had CNS VZV and TBM infections, respectively, both of the positive detection of *Aspergillus* spp. was considered clinically important (Chen J. et al., 2021). The authors did not provide additional microbiological evidence to support CNS *Aspergillus* spp. infection, and therefore whether *Aspergillus* spp. truly caused CNS disorder was unclear. At this point, it is difficult to completely avoid the risk of involving additional unnecessary diagnostic investigations and unnecessary treatment associated with the application of mNGS.

Our study was limited by retrospective design and relatively low sample size for specific CNS infection. The calculated sensitivity and specificity of CSF mNGS for diagnosing CNS infection should be interpreted with prudence. Due to the shortage of commercial diagnostic kits and the CSF sample volume restriction, it was not possible for us to validate every mNGS report with pathogen-specific PCR or other certified traditional microbiological tests. For those reasons, interpretation of the mNGS results is still challenging. Finally, the unbiased mNGS in our study only targeted the DNA elements mainly due to the high cost of mNGS, and therefore it may miss the identification of some RNA virus in the CSF samples. Whether adding RNA sequencing (for example, the commercially available PMseq-RNA) to improve the diagnostic performance needs to be further explored. Despite those limitations, the finial diagnoses were clear for the vast majority (> 90%) of the cases when CSF mNGS was integrated into diagnostic panel. This finding suggests that CSF mNGS is a promising microbiological diagnostic test for CNS infections in HIV-infected patients.

Metagenomics sequencing analysis is a “finding the needle in a haystack” screening technique and may be the most complex among all known laboratory-developed tests. It is a long road to standardization in a real clinical context, but to achieve better quality and higher reproducibility, efforts could be made in simple and easy wet-lab manipulations, for example, using library prep/PCR in one-tube techniques. Barcoding the library samples with unique dual-index combinations to minimize cross-talking, especially to protect those uncertain samples from reads generated by significant infectious samples. Make sure process controls are always included to further examine “kitome,” and “splashome” contamination batch by batch. It is recommended to monitor the variations of lab environmental microbiomes (air, water, surface, etc.) in the mid or long-term. Additionally, to improve the test’s predictive value, a well-curated clinical microbe’s database and joint interpretation by an expert panel (lab technicians, bioinformaticians, microbiologists, pathologists, and clinical physicians) are also needed.

In conclusion, CSF mNGS is a very sensitive tool for detecting common opportunistic CNS pathogen in HIV-infected patients; however, its performance in CNS tuberculosis is unsatisfactory. CMV and EBV are commonly detected by CSF mNGS from CSF samples in HIV-infected patients, the clinical importance of which is likely to be associated with the burden of the viruses. Clinical interpretation of CSF mNGS results is still challenging because of sample cross-contamination, environmental microbial contamination and lack of consensus standard for distinguishing carrier state/colonization and infection. Those obstacles need to be addressed in future studies.

## Data availability statement

The original contributions presented in the study are included in the article/Supplementary material; further inquiries can be directed to the corresponding authors.

## Author contributions

ZH and ZP conceived and designed the study. YunZ, WZ, XY, and XL collected the data. CC, YC, HW, and ZH were involved in clinical interpretation of mNGS results and the clinical management of the patients. YunZ, WZ, XY, YuaZ, XW, and YW analyzed the data and drafted the manuscript. All authors contributed to the article and approved the submitted version.

## Funding

This study was funded in part by the National Key Research and Development Program of China (2019YFC1200500 and 2019YFC1200501) and Nanjing Medical Science and Technique Development Foundation (ZKX21037). The funders had no role in study design, data collection and analysis, decision to publish, or preparation of the manuscript.

## Conflict of interest

The authors declare that the research was conducted in the absence of any commercial or financial relationships that could be construed as a potential conflict of interest.

## Publisher’s note

All claims expressed in this article are solely those of the authors and do not necessarily represent those of their affiliated organizations, or those of the publisher, the editors and the reviewers. Any product that may be evaluated in this article, or claim that may be made by its manufacturer, is not guaranteed or endorsed by the publisher.

## Footnotes

^1^ ftp://ftp.ncbi.nlm.nih.gov/genomes/

^2^ www.r-project.org
