# Supplementary material for: Metagenomic next-generation sequencing for identification of central nervous system pathogens in HIV-infected patients
Source: Front Microbiol. 2022 Nov 15;13:1055996. doi: 10.3389/fmicb.2022.1055996 (PMC9705764; doi:10.3389/fmicb.2022.1055996)
Supplement: Supplementary file 1 [file Table_1.docx]

**Supplement Table 1** **2×2 cross-tabulations for calculating diagnostic performance of mNGS^1^.**

A mNGS for diagnosing EBV-associated CNS disorders including CNS lymphoma and encephalitis

|  | | **Final composite diagnosis** | |
| --- | --- | --- | --- |
|  |  | negative | positive |
| **mNGS** | negative | 35 | 0 |
|  | positive | 29^2^ | 10 |

B mNGS for diagnosing CMV encephalitis

|  | | **Final composite diagnosis** | |
| --- | --- | --- | --- |
|  |  | negative | positive |
| **mNGS** | negative | 50 | 0 |
|  | positive | 13^2^ | 11 |

C mNGS for diagnosing PML

|  | | **Final composite diagnosis** | |
| --- | --- | --- | --- |
|  |  | negative | positive |
| **mNGS** | negative | 60 | 1 |
|  | positive | 0 | 13 |

D mNGS for diagnosing CNS tuberculosis

|  | | **Final composite diagnosis** | |
| --- | --- | --- | --- |
|  |  | negative | positive |
| **mNGS** | negative | 67 | 3 |
|  | positive | 2 | 2 |

E mNGS for diagnosing Toxoplasma encephalitis

|  | | **Final composite diagnosis** | |
| --- | --- | --- | --- |
|  |  | negative | positive |
| **mNGS** | negative | 68 | 0 |
|  | positive | 0 | 6 |

F mNGS for diagnosing Cryptococcal meningitis

|  | | **Final composite diagnosis** | |
| --- | --- | --- | --- |
|  |  | negative | positive |
| **mNGS** | negative | 66 | 0 |
|  | positive | 0 | 8 |

mNGS, metagenomic next-generation sequencing; EBV, Epstein–Barr virus; CNS, central nervous system; CMV, cytomegalovirus; PML, progressive multifocal leukoencephalopathy;

1, For the purpose of illustration, all 6 patients with undetermined causes of neurological symptoms (including one patient with 1 Mycobacterium tuberculosis sequence identified by mNGS but deemed to not have tuberculosis; 3 detections of EBV without clinical significance) had been excluded from analysis. Therefore, diagnostic performance of CSF mNGS were calculated from 74 samples. The final composite diagnosis was used as the reference standard.

2, Although virus sequences were identified by mNGS, the patients were not though to have related CNS diseases because the clinical condition improved without targeted treatment.
